# Accurate Prediction of Stroke for Hypertensive Patients Based on Medical Big Data and Machine Learning Algorithms: Retrospective Study

**DOI:** 10.2196/30277

**Published:** 2021-11-10

**Authors:** Yujie Yang, Jing Zheng, Zhenzhen Du, Ye Li, Yunpeng Cai

**Affiliations:** 1 Shenzhen Institute of Advanced Technology Chinese Academy of Sciences Shenzhen China; 2 University of Chinese Academy of Sciences Beijing China; 3 Shenzhen Health Information Center Shenzhen China; 4 Joint Engineering Research Center for Health Big Data Intelligent Analysis Technology Shenzhen China

**Keywords:** stroke, medical big data, electronic health records, machine learning, risk prediction, hypertension

## Abstract

**Background:**

Stroke risk assessment is an important means of primary prevention, but the applicability of existing stroke risk assessment scales in the Chinese population has always been controversial. A prospective study is a common method of medical research, but it is time-consuming and labor-intensive. Medical big data has been demonstrated to promote disease risk factor discovery and prognosis, attracting broad research interest.

**Objective:**

We aimed to establish a high-precision stroke risk prediction model for hypertensive patients based on historical electronic medical record data and machine learning algorithms.

**Methods:**

Based on the Shenzhen Health Information Big Data Platform, a total of 57,671 patients were screened from 250,788 registered patients with hypertension, of whom 9421 had stroke onset during the 3-year follow-up. In addition to baseline characteristics and historical symptoms, we constructed some trend characteristics from multitemporal medical records. Stratified sampling according to gender ratio and age stratification was implemented to balance the positive and negative cases, and the final 19,953 samples were randomly divided into a training set and test set according to a ratio of 7:3. We used 4 machine learning algorithms for modeling, and the risk prediction performance was compared with the traditional risk scales. We also analyzed the nonlinear effect of continuous characteristics on stroke onset.

**Results:**

The tree-based integration algorithm extreme gradient boosting achieved the optimal performance with an area under the receiver operating characteristic curve of 0.9220, surpassing the other 3 traditional machine learning algorithms. Compared with 2 traditional risk scales, the Framingham stroke risk profiles and the Chinese Multiprovincial Cohort Study, our proposed model achieved better performance on the independent validation set, and the area under the receiver operating characteristic value increased by 0.17. Further nonlinear effect analysis revealed the importance of multitemporal trend characteristics in stroke risk prediction, which will benefit the standardized management of hypertensive patients.

**Conclusions:**

A high-precision 3-year stroke risk prediction model for hypertensive patients was established, and the model's performance was verified by comparing it with the traditional risk scales. Multitemporal trend characteristics played an important role in stroke onset, and thus the model could be deployed to electronic health record systems to assist in more pervasive, preemptive stroke risk screening, enabling higher efficiency of early disease prevention and intervention.

## Introduction

Stroke is the third leading cause of death globally, and China has become the country with the highest lifetime risk of stroke (39.3%) worldwide [1,2]. According to the China Stroke Report 2019, stroke has been the leading cause of death and disability among Chinese adults, and the incidence shows a younger trend. In 2018, the number of deaths from cerebrovascular diseases reached 1.57 million, accounting for 22% of the deaths of Chinese residents [3]. The damage caused by stroke is often irreversible, and stroke is prone to recur, with an annual recurrence rate of 3%-5%, and the condition aggravates with the increasing number of recurrences. However, stroke is preventable and controllable, and early intervention of modifiable risk factors can effectively reduce the occurrence and death of stroke [4].

The pathogenesis of stroke is complicated and often results from the synergistic effect of various risk factors [5]. The known risk factors include gender, age, race, hypertension, diabetes, hyperlipidemia, systolic blood pressure (SBP), smoking, atrial fibrillation, etc. In recent years, studies have been discovering or proposing new risk factors of stroke, such as lipoprotein [6], triglyceride-glucose index [7], obstructive sleep apnea [8], vascular profile [9], heart failure [10], sleep disturbances [11], cerebral microbleeds [12], diet [13], imaging biomarkers [14], genetics [15], and environment [16].

Stroke risk assessment is an effective means to identify high-risk groups, and various well-known risk assessment scales have been established, such as the Framingham Stroke Risk Profile (FSRP) [17,18], SCORE-based fatal cardiovascular disease risk model [19,20], QStroke [21], pooled cohort risk equation (PCE) for atherosclerotic cardiovascular disease (ASCVD) [22,23], CHADS2 [24], CHA2DS2-VASc [25], HAS-BLED [26], and ATRIA [27]. However, the risk factors for stroke vary slightly by region and race [28,29], and these scales are mostly based on European and American populations, which tend to overestimate the risk of the Chinese population [30,31]. Some scales, such as the acute cardiovascular events risk model based on the Chinese Multiprovincial Cohort Study (CMCS) [32], the ASCVD risk model based on the China-PAR project [33,34], and a stroke risk model among adults in Taiwan [35], have also been estimated based on the Chinese population, but they have not been widely used. Moreover, these models are established based on long-term prospective studies, which are time-consuming and labor-intensive.

With the widespread application of electronic medical record (EMR) systems, a massive amount of medical data have been accumulated, which provides a fast, cost-efficient approach to collecting large-scale samples for retrospective studies. Medical big data has been demonstrated to promote medical applications such as discovering disease risk factors and prognosis, but it has also attracted extensive concerns [36-38]. Retrospective studies based on EMRs face enormous challenges, the most important of which is a large amount of missing data. How to construct effective features, especially those of medical significance, is crucial to building high-precision risk models, and the prevalence of machine learning provides interesting tools to optimize the modeling process.

**Figure 1:**
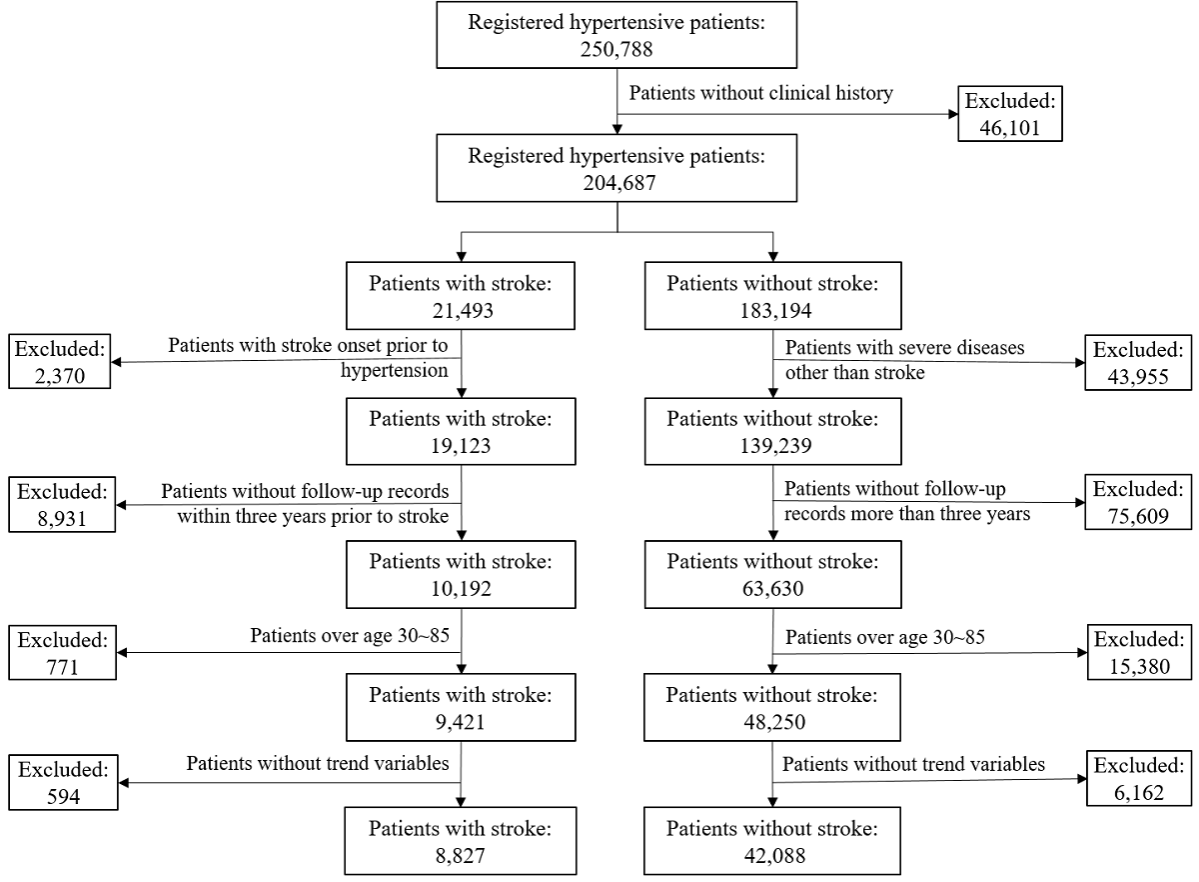
The screening process of study population.

In this study, we started from the substantial historical stock EMRs of registered hypertensive patients in Shenzhen and aimed to establish a high-precision stroke risk prediction model through medical big data and machine learning. A total of 250,788 registered hypertensive patients were collected, of which 21,493 developed stroke during the 3-year follow-up. After strict screening, only 57,671 samples were selected for risk modeling, as shown in Figure 1. We constructed characteristics from the multitemporal EMRs, established 3-year stroke risk prediction models based on 4 machine learning algorithms, and compared performance with well-known risk assessment scales. Finally, we analyzed the nonlinear correlation between continuous variables and the occurrence of stroke. Our study revealed the important role of multitemporal trend characteristics in improving the performance of stroke risk prediction models, which will benefit the standardized management of hypertensive patients.

## Methods

### Data Resource and Study Population

The data used in this study are the electronic health records from the Shenzhen Health Information Big Data Platform, which has access to more than 4000 health institutions, including 85 hospitals and over 650 community health service centers. The platform covered medical service records, including disease management, outpatient service, hospitalization, laboratory test, imaging examination, and physical examination. Disease management covers patients with hypertension, diabetes, cancer, etc, who are registered and regularly followed up. At present, the platform has more than 600 million EMRs from 2010 to 2020. Medical records among different institutions of the same patient can be associated with a unique personal identification number. Since medical records were collected in routine clinical activities, patients had agreed and authorized their use during the consultation process. According to the Guidelines of the WMA Declaration of Helsinki, the study was approved by the SIAT IRB (SIAT-IRB-151115-H0084).

Hypertension is the primary risk factor for stroke. Moreover, hypertensive patients are the key population of disease management, and thus long-term physical examination results have been accumulated, which are essential data for stroke risk prediction. This study focused on registered hypertensive patients and aimed to establish a high-precision stroke risk prediction model. A total of 250,788 hypertensive patients were collected from the platform, with an average follow-up of 4.5 years. The stroke diagnosis was extracted from the main diagnosis fields of the outpatient or inpatient records according to the International Statistical Classification of Diseases and Related Health Problems 10th Revision diagnostic codes [39], including I60 (subarachnoid hemorrhage), I61 (intracerebral hemorrhage), I62 (other nontraumatic intracranial hemorrhages), I63 (cerebral infarction) and I64 (stroke, not specified as hemorrhage or infarction), and excluded I69 (sequelae of cerebrovascular disease). Finally, there were 21,493 cases of stroke onset, and the date of the first occurrence of a stroke diagnosis in the clinical records was taken as the date of stroke diagnosis.

We limited the study to patients with at least one outpatient or hospitalization record to ensure the reliability of outcomes, and thus 46,101 patients were excluded. We excluded patients with stroke (positive cases), those with stroke prior to hypertension, and those without follow-up records within 3 years before stroke onset. In addition, patients without stroke (negative cases), those with heart disease, renal failure, or tumor, and those without more than 3 years of follow-up records were also excluded. In addition, patients were limited to 30-85 years old. As a result, 57,671 hypertensive patients were included in the study, of which 9421 patients had a stroke within 3 years of follow-up. Moreover, patients were required to have trend change variables (eg, mean SBP), and thus 6756 patients were excluded. The detailed screening process of the study population is shown in Figure 1.

### Feature Extraction

The medical records of 57,671 samples were extracted from the platform, including resident information, lifestyle, family history, follow-up records with registered hypertensive patients, outpatient and hospitalization records, and laboratory test results. Medical records were collected from hundreds of health institutions with slightly different medical service systems, resulting in diverse data formats, poor data quality, and even a large number of missing fields. We first performed a series of cleaning operations on the medical records, including deleting outliers or replacing them with null values, unit unification of test results, and drug classification.

Given this is a retrospective study based on real-world multitemporal medical data, the event endpoint and baseline needed to be predefined before feature extraction. For positive cases, the endpoint was the date of stroke diagnosis, and baseline was defined as the date of the first follow-up record within 3 years prior to the endpoint. For negative cases, the endpoint was the date of the last medical service record, and baseline was defined as the date of the last follow-up record 3 years before the endpoint. The physiological parameters in the follow-up record at baseline were extracted as characteristics, such as age, SBP, diastolic blood pressure (DBP), pulse pressure difference (PPD; the difference between SBP and DBP), heart rate (HR), BMI, glucose.

Secondly, trend characteristics of physiological parameters based on multitemporal follow-up records before baseline were specially constructed, such as SBP, DBP, PPD, HR, BMI, and glucose. The follow-up records were grouped by patients and sorted in ascending order of follow-up date, and the difference in the two consecutive records was calculated, which were marked with *_delta. Moreover, the maximum, minimum, mean, and derivation of physiological parameters of each patient and their differences were calculated.

Thirdly, historical symptoms were extracted from the outpatient and hospitalization records before baseline. In this study, only some symptoms that are potentially associated with stroke attack were extracted, such as diabetes, hyperlipemia, sleep disorder, etc. Moreover, demographic characteristics (ie, gender), family disease history (ie, family history of coronary heart disease or FAM_CHD), lifestyles (ie, smoking and drinking), and drug categories (ie, antihypertensive drug use) were extracted. The features were binarized based on their existence.

Finally, laboratory test results were extracted. According to statistics, less than 10% of patients had laboratory test records near the baseline. For the purpose of comparing model prediction performance with existing scales, only necessary blood lipid tests were extracted, including triglycerides, total cholesterol, low-density lipoprotein cholesterol, and high-density lipoprotein cholesterol (HDL-C).

Proper feature selection is beneficial to improve the performance of the model. First, features with missing values above 30% were removed. Then, correlation analysis and univariate trend analysis were adopted to remove redundant features, and a two-tailed *P* value <.05 was considered a significant correlation. In addition, some features of existing research were manually retained.

### Prediction Modeling

An ensemble method extreme boosting gradient (XGBoost) [40] was used to establish a 3-year stroke risk prediction model for hypertensive patients and compared with the other 3 widely-used traditional machine learning algorithms, including logistic regression [41], support vector machine (SVM) [42], and random forest [43].

XGBoost is an integration algorithm based on multiple decision trees under the gradient boosting framework. Unlike traditional gradient boosting decision trees, XGBoost supports column sampling, which can reduce overfitting and calculation. In addition, XGBoost considers a sparse matrix and can automatically learn its splitting direction for samples with missing values.

Logistic regression is a classical classification algorithm widely used in epidemiology and medicine, such as risk factor discovery, disease risk prediction, and automatic disease diagnosis. Logistic regression is a generalized linear regression model that introduces the sigmoid function to normalize dependent variables, thus making it more focused on the classification boundaries and increasing its robustness.

SVM is a bicategorical algorithm, which is characterized by the ability to minimize empirical errors and maximize geometric edge regions at the same time. SVM also includes nuclear techniques, which makes it a substantial nonlinear classifier. In addition, the stability and sparsity of SVM give it good generalization capability.

Random forest is also an ensemble algorithm based on decision trees, which determines the final prediction by combining the outcome of multiple weak classifiers. In random forests, the base classifiers are trained independently, so the learning process is very fast. Moreover, random forests have the advantages of evaluating the importance of variables and resisting overfitting and supporting column sampling and missing values by default.

According to a ratio of 7:3, we randomly divided the data set into training and test sets with balanced positive and negative cases. We performed 5-fold cross-validation on the training set and validated the performance of the models on the test set. Five evaluation criteria were used to validate the models, including the area under the receiver operating characteristic curve (AUC), accuracy, recall, specificity, and F1-score. For continuous features, the missing values were filled with the mean of each feature, and the data were standardized by the mean and variance of the feature.

All the experiments were performed under the environment manager Anaconda of the Linux server in the isolated intranet, and a Python3.6.5 kernel was used for data processing and modeling. We implemented 4 algorithms using the Scikit-learn library in the Python programming environment [44].

## Results

### Characteristics Description

**Figure 2:**
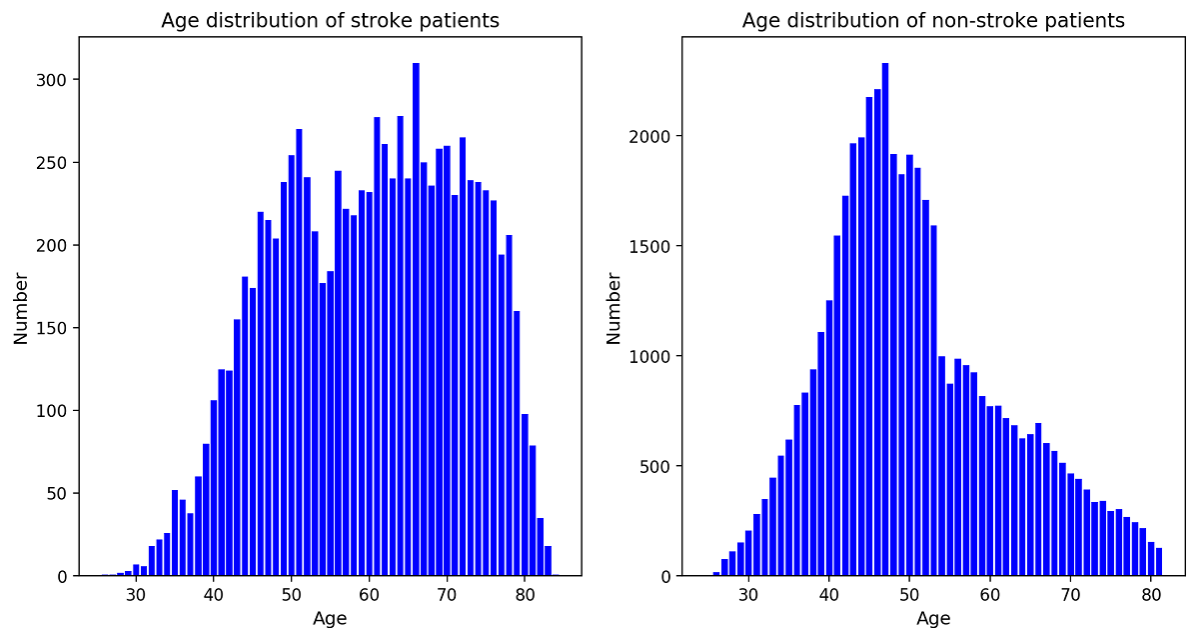
Age distribution of stroke and nonstroke patients.

A total of 50,915 registered patients with hypertension were screened into the study cohort, and 8827 patients developed stroke within 3-year follow-up. In the study cohort, the positive/negative ratio was about 1:4.7, and the age distribution was different, as depicted in Figure 2.

In order to balance positive and negative cases, we performed a random stratified sampling of negative cases according to gender ratio and age stratification of positive cases. Age was stratified into 30 to 40, 40 to 50, 50 to 60, 60 to 70, and 70 to 85 years, and the proportion of negative to positive cases in gender and age stratification was calculated. We took the minimum proportion as the sampling rate and randomly selected the corresponding number of samples from the negative cases of each group. After stratified sampling, 11,126 negative cases and 8827 positive cases were used for modeling, and the gender and age distribution are depicted in Table 1.

**Table 1:** Gender and age distribution before and after stratified sampling.

Characteristics		Positive cases (N=8,827), n (%)	Negative cases (N=42,088), n (%)	Negative cases after sampling N=11,126), n (%)
Gender, male		5251 (59.49)	25990 (61.75)	6174 (55.49)
**Age, years**				
	30-40	414 (4.69)	5843 (13.88)	522 (4.69)
	40-50	1746 (19.78)	17342 (41.20)	2204 (19.81)
	50-60	2104 (23.84)	10415 (24.75)	2656 (23.87)
	60-70	2462 (27.89)	5448 (12.94)	3108 (27.93)
	70-85	2088 (23.65)	2636 (6.26)	2636 (23.69)

A total of 77 features were extracted from the medical records, and eventually, 49 features were used as input for the machine learning algorithms. Blood lipid test results were not included because the missing ratio was more than 80%. Table 2 shows the statistical distribution of partial features of higher correlation (*P* value less than .01).

**Table 2:** Distribution of the basic characteristics.

	Characteristics	Positive cases (N=8,827)	Negative cases (N=11,126)	*P* value^a^
**Demographics**				
	Gender, n (%), male	5,251 (59.49)	6174 (55.49)	<.001
	Age, mean (SD), years	60.21 (11.88)	59.73 (11.94)	.005
	Years_after_hypertension, mean (SD), years	6.25 (5.64)	6.78 (5.27)	<.001
**Lifestyle (current or previous), n (%)**				
	Smoking	768 (8.70)	1233 (11.08)	<.001
	Drink	1000 (11.33)	1643 (14.77)	<.001
**Family history, n (%)**				
	FAM_hypertension	239 (2.71)	489 (4.40)	<.001
	FAM_diabetes	57 (0.65)	116 (1.04)	.002
**Physical examination, mean (SD)**				
	SBP^b^, mmHg	133.76 (13.42)	131.33 (10.02)	<.001
	DBP^c^, mmHg	81.93 (9.56)	80.17 (7.45)	<.001
	PPD^d^, mmHg	52.16 (10.59)	51.15 (8.81)	<.001
**Trend characteristics, mean (SD)**				
	N_followup_1year	4.13 (3.66)	5.89 (3.84)	<.001
	SBP_max, mmHg	140.29 (14.56)	142.77 (13.46)	<.001
	SBP_min, mmHg	127.17 (14.09)	122.81 (10.21)	<.001
	SBP_mean mmHg	133.20 (11.58)	131.75 (7.90)	<.001
	DBP_max, mmHg	86.47 (9.69)	89.10 (8.51)	<.001
	DBP_min, mmHg	76.67 (10.16)	73.42 (7.36)	<.001
	DBP_mean, mmHg	81.35 (8.17)	80.71 (5.80)	<.001
	PPD_max, mmHg	58.41 (12.02)	61.48 (10.83)	<.001
	PPD_min, mmHg	46.01 (11.05)	41.69 (8.28)	<.001
	PPD_mean, mmHg	51.89 (8.75)	51.04 (6.26)	<.001
	HR^e^_max, times/min	78.57 (7.08)	79.57 (7.11)	<.001
	HR_min, times/min	74.29 (6.68)	72.97 (5.97)	<.001
	SBP_delta_mean, mmHg	4.37 (3.53)	4.01 (3.17)	<.001
	DBP_delta_mean, mmHg	3.46 (2.44)	3.24 (2.10)	<.001
	PPD_delta_mean, mmHg	4.30 (3.08)	4.04 (2.68)	<.001
	HR_delta_mean, times/min	1.23 (1.83)	1.08 (1.51)	<.001
**Medical history,** **n** **(%)**				
	Prior cardiovascular diseases	176 (1.99)	11 (0.1)	<.001
	Atrial fibrillation	53 (0.6)	16 (0.14)	<.001
	Atherosclerosis	488 (5.53)	358 (3.22)	<.001
	sleep disorder	99 (1.12)	475 (4.27)	<.001
	Dizziness and headache	1094 (12.39)	1804 (16.21)	<.001
	Malaise and fatigue	6 (0.07)	55 (0.49)	<.001
	Giddiness	9 (0.10)	55 (0.49)	<.001
	Migraine	7 (0.08)	38 (0.34)	<.001
	Antihypertensive treatment	8551 (96.87)	10905 (98.01)	<.001
	Lipid-lowering drug	1123 (12.72)	1046 (9.40)	<.001

^a^Pearson chi-square test was applied.

^b^SBP: systolic blood pressure.

^c^DBP: diastolic blood pressure.

^d^PPD: pulse pressure difference.

^e^HR: heart rate.

### Predictive Performance Evaluation

**Figure 3:**
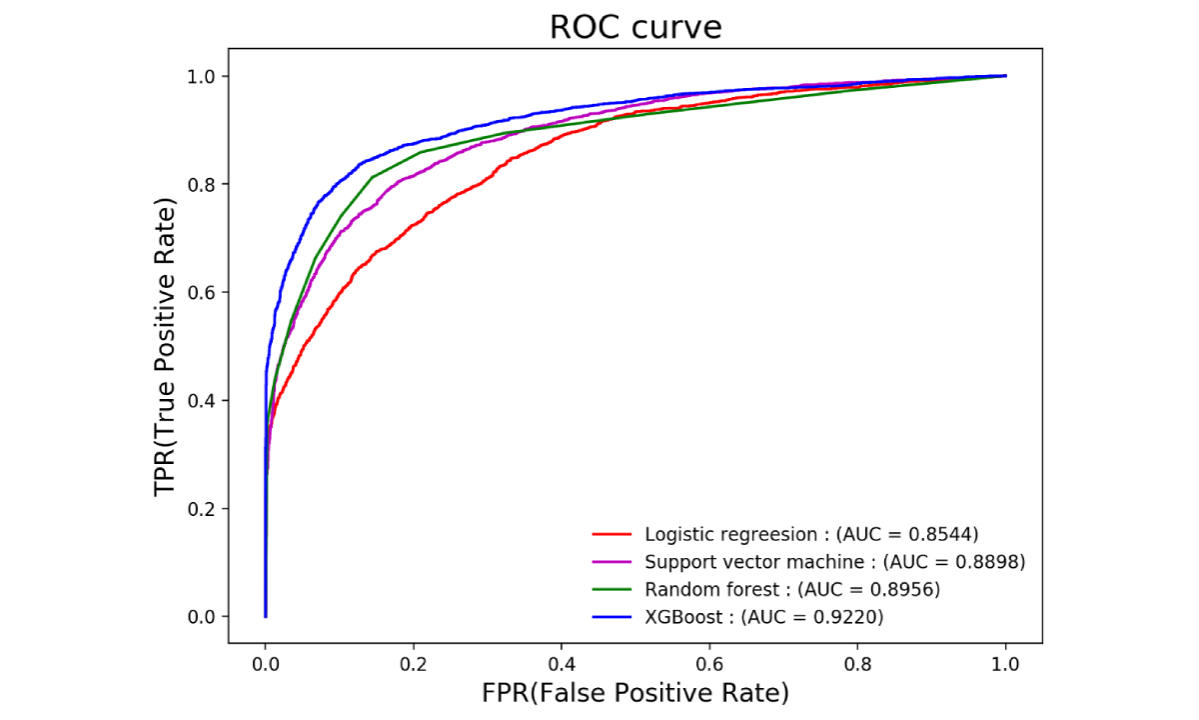
The receiver operating characteristic curve of the four algorithms.

According to the ratio of 7:3, the data set was randomly divided into a training set (N=13,967) and test set (N=5986), and the ratio of positive to negative cases was balanced (ratio=1:1.26). Table 3 shows the performance of the 4 algorithms on the test set. The tree-integration algorithm XGBoost achieved the best performance with AUC of 0.9220, followed by random forest with AUC of 0.8956. Logistic regression had the worst performance with AUC of 0.8544, as shown intuitively from the receiver operating characteristic (ROC) curve in Figure 3.

**Table 3:** Model performance of four different algorithms.

Methods	AUC^a^	Accuracy	Recall	F1-score	Specificity
Logistic regression	0.8544	0.7726	0.7141	0.7354	0.8191
SVM^b^	0.8898	0.8112	0.7844	0.7861	0.8325
Random forest	0.8956	0.8343	0.8157	0.8133	0.8490
XGBoost^c^	0.9220	0.8478	0.8512	0.8319	0.8451

^a^AUC: area under the receiver operating curve.

^b^SVM: support vector machine.

^c^XGBoost: extreme gradient boosting.

### Features Importance

**Figure 4:**
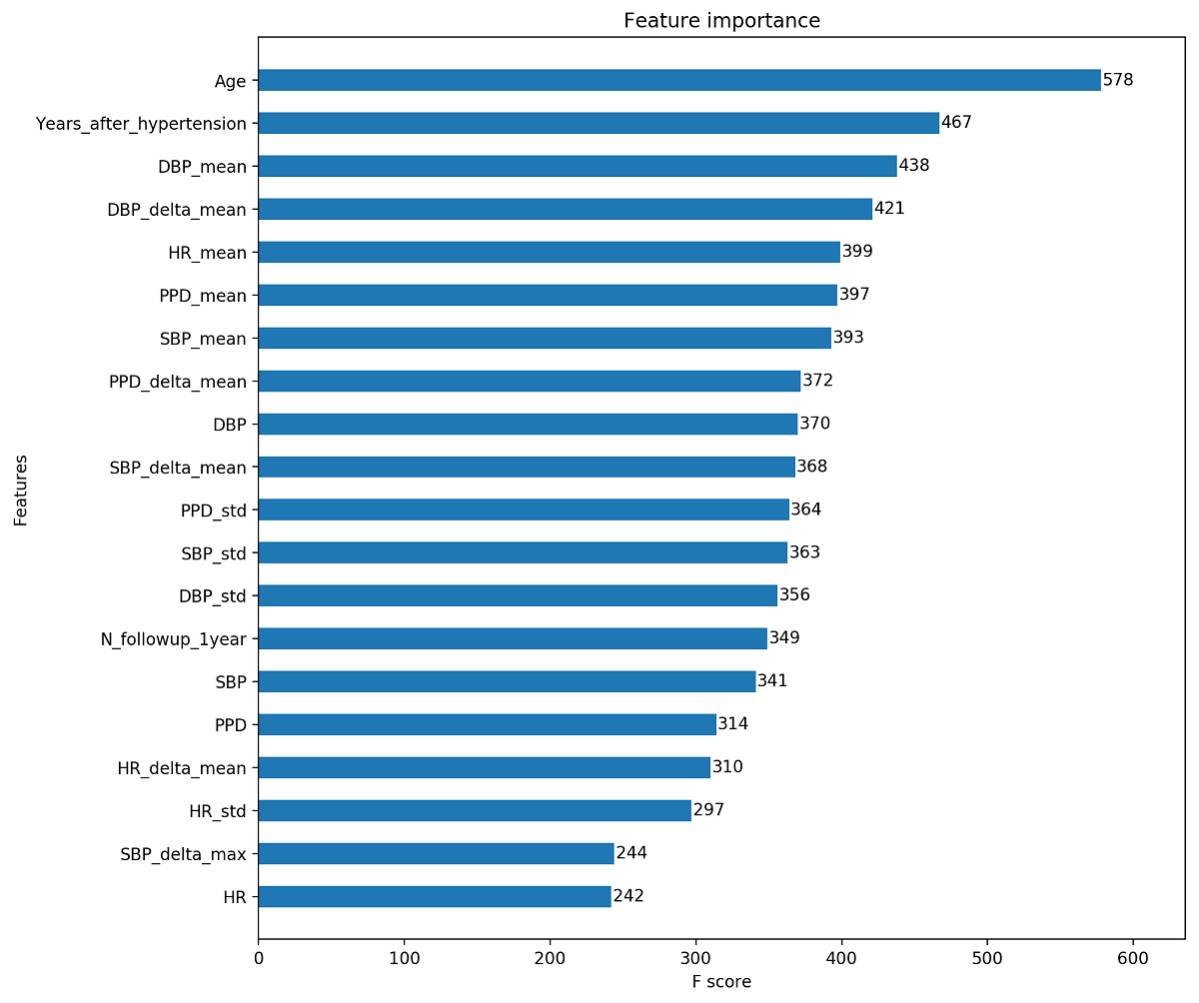
Features of the top 20 importance in XGBoost model. DBP: diastolic blood pressure; HR: heart rate; PPD: pulse pressure difference; SBP: systolic blood pressure; XGBoost: extreme gradient boosting.

Feature importance measures the relative contribution of the features to modeling. The top 20 features are depicted in Figure 4. In addition to the traditional risk factors contained in well-known scales, the trend characteristics of physiological parameters also played an important role in modeling, such as PPD, HR_mean, and PPD_delta_mean. The feature PPD could reflect the change of vascular elasticity, and when PPD is too large or too small, the disease's hidden danger would be indicated and should be addressed. In addition, the mean of the difference between 2 adjacent follow-up records could reflect the control level of physiological parameters, which are easily obtained in daily monitoring and promote the health management of hypertensive patients.

### Nonlinear Effects of Continuous Features

**Figure 5:**
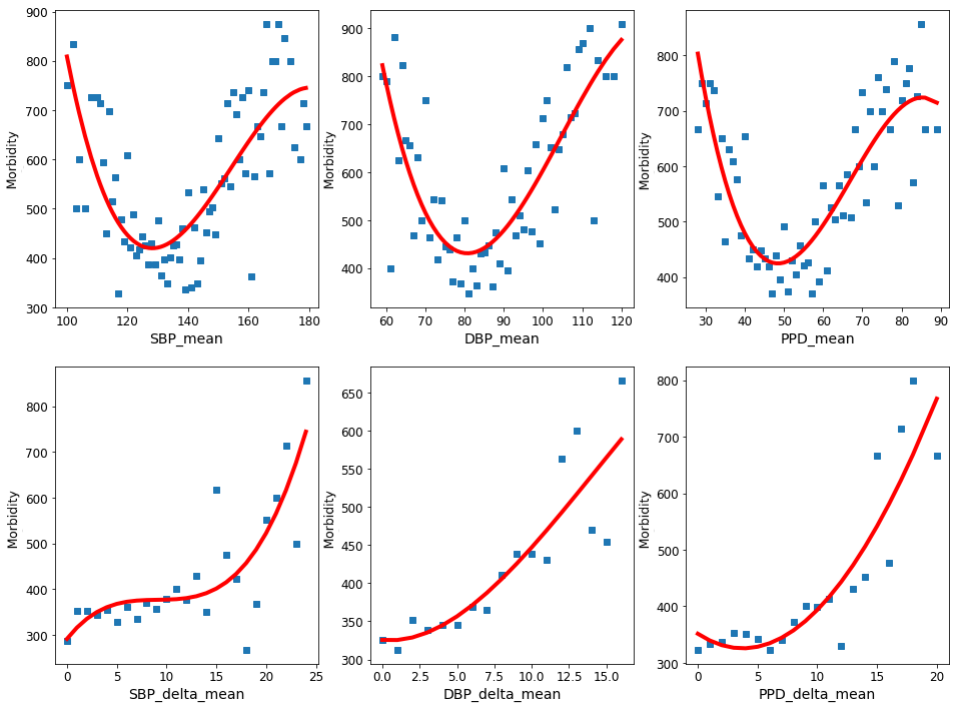
Nonlinear effect of six continuous features on the morbidity of stroke. DBP: diastolic blood pressure; PPD: pulse pressure difference; SBP: systolic blood pressure.

We performed a univariate trend analysis of continuous features based on the 3-year risk prediction data set to analyze the effect of characteristics on stroke occurrence further. Morbidity was defined as the number of stroke cases in a thousand samples under a characteristic value, and the relationship between the morbidity and characteristic values was fitted. In this study, we chose Gaussian, polynomial, and exponential functions to fit the curve, and the fitting effect was evaluated by discriminant coefficient *R*^2^ [45]. Figure 5 showed the nonlinear effects of 6 features, which were the top modifiable risk factors in the feature importance of Figure 4. We found that the effect of some factors (eg, SBP_mean, DBP_mean, HR_mean, and PPD_mean) formed a U-shaped trend, where the marginal risk was minimized when the factor fell within a given range while increasing both when it went lower or higher. Unsurprisingly, the turn-points for the 3 factors were highly consistent with the blood pressure control targets of the latest hypertension guidelines. On the other hand, the effects of DBP_delta_mean and PPD_delta_mean formed a hinge-like sharp, which revealed the importance of stable blood pressure for stroke prevention in hypertension patients.

## Discussion

### Principal Findings

We had developed a high-precision risk prediction model of stroke for hypertensive patients based on large-scale electronic health records from a regional medical information platform and validated the prediction performance on an independent test set. The integrated tree-based XGBoost algorithm achieved the best prediction performance with an AUC of 0.9220 and outperformed the other 3 traditional algorithms. Besides the traditional risk factors, such as age, gender, SBP, smoking, diabetes, and antihypertensive drug use, we specially constructed several changing-trend variables from multitemporal medical records, which were confirmed to be nonlinearly correlated with stroke onset. The effect of nonlinear correlation justified the necessity of adopting sophisticated nonlinear machine learning models over traditional linear regressions. Furthermore, with nonlinear ensemble algorithms such as XGBoost used in this study, there was no need to select variables in advance even when the number of potential variables was large, which was different from most traditional clinical studies and enabled the identification of novel biomarkers with both linear and nonlinear effects during modeling process through mining large-scale population data. This was an advantage brought by big data technologies.

### Comparison With Traditional Statistical Models

Several risk models based on long-period prospective studies have been widely used to screen high-risk populations, such as Framingham studies, QStroke, and PCE. Considering the target events and wide application of the models, we selected to compare the model's performance based on XGBoost with the revised FSRP [20] and CMCS risk scale [32].

The FSRP, originally described in 1991 [19], had been validated in other cohorts, was recommended by the American Heart Association. The study population was between 55 and 84 years old. However, the profile had been demonstrated by several studies to overestimated risk; therefore, the profile was updated in 2017. The revised FSRP better predicted current stroke risk in 3 large community samples, integrating gender, age, current smoking habits, prevalent cardiovascular disease (including myocardial infarction, angina, coronary insufficiency, intermittent claudication, and congestive heart failure), atrial fibrillation, diabetes, SBP, and antihypertensive treatment. Moreover, the profile provided a multiyear prediction model for 10 years. In this study, we selected the 3-year and 10-year models to compare the performance.

The CMCS risk scale was a 10-year risk prediction model of acute cardiovascular event (acute coronary heart disease and acute stroke) proposed in 2003. The study population was aged 35 to 64 years living in 11 provinces and cities of China. The risk factors used in the model included gender, age, diabetes, smoking, SBP, total cholesterol, and HDL-C.

**Figure 6:**
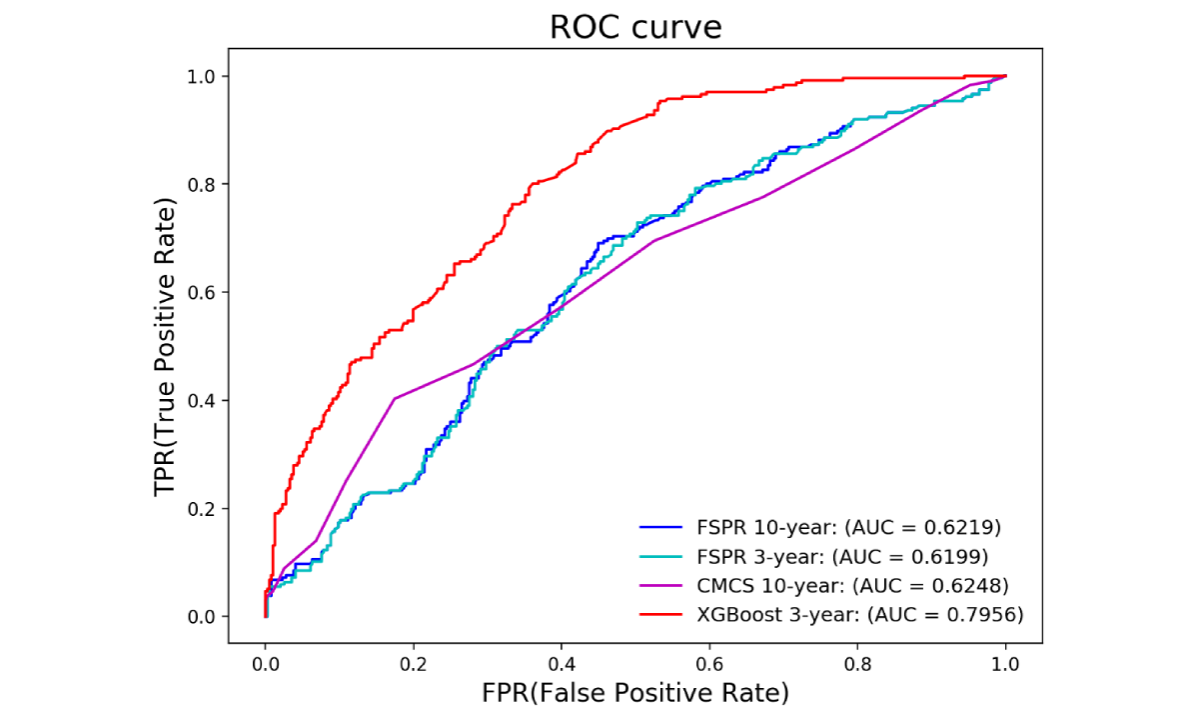
Receiver operating characteristic curve compared with three traditional risk scales. AUROC: area under the receiver operating characteristic; CMCS: Chinese Multi-provincial Cohort Study; FSRP: Framingham Stroke Risk Profile; XGBoost: extreme gradient boosting.

We screened a subset of 632 samples from 76,494 samples that simultaneously met the FSRP and CMCS profile, of which 236 had stroke onset. These samples were assigned to the test set in the first step of our model-building process. Figure 6 depicts the ROC curve achieved by the 4 models. The developed model based on XGBoost achieved a higher performance with an AUC of 0.7956, and there was no significant difference between the other 3 scales.

### Limitations and Future Research

This work was a retrospective study based on historical stock data collected at different periods. There were a large number of missing values in characteristic variables, which may affect the sample population size and the performance of the model. In addition, due to the insufficiency of laboratory test results, the established model did not include the biochemical indicators in the traditional scales, such as TC and HDL-C. However, the impact of missing information was equal for both the positive and negative cases so that no significant biases were likely to be introduced through missing data. Compared with the benefits obtained by the enlarged population and the abundance of clinical features, the data's increased noise was considered acceptable. In addition, the study cohort was imbalanced in view of the numbers of positive cases and negative cases. We performed randomly stratified sampling according to gender ratio and age stratification, which may not represent the rest of the patients accurately. We are currently accumulating longer periods of medical data as well as a larger population and trying to further validate and improve the model with recent data.

### Conclusions

We established a high-precision 3-year stroke risk prediction model for hypertensive patients based on large-scale EMRs and verified that the proposed model could perform better than traditional risk scales. In addition, the features in the model are routinely accessible data, so the model could be easily implemented in EMR systems to help with a more pervasive, preemptive screening of stroke risk, enabling higher efficiency of early disease prevention and intervention.

## Abbreviations

ASCVD: atherosclerotic cardiovascular disease
AUC: area under the ROC curve
CMCS: Chinese Multiprovincial Cohort Study
DBP: diastolic blood pressure
EMR: electronic medical record
FSRP: Framingham Stroke Risk Profile
HDL-C: high-density lipoprotein cholesterol
HR: heart rate
PCE: pooled cohort risk equation
PPD: pulse pressure difference
ROC: receiver operating characteristic
SBP: systolic blood pressure
SVM: support vector machine
TC: total cholesterol
XGBoost: extreme gradient boosting
